# Support interventions for nurses working in acute psychiatric units: A systematic review

**DOI:** 10.4102/hsag.v27i0.1811

**Published:** 2022-04-29

**Authors:** Ntombiyakhe Bekelepi, Penelope Martin

**Affiliations:** 1 School of Nursing, Faculty of Community Health Science, University of the Western Cape, Cape Town, South Africa

**Keywords:** acute mental health, emotional support, intervention programme, nurse, mental health unit, psychiatric hospital, supportive intervention, workplace violence

## Abstract

Diverse support interventions have been implemented to provide support for nurses working in acute psychiatric settings. These interventions aimed at modifying the psychological and social factors, as they either prevent stress responses or reduce its effects to improve the psychological well-being of staff. This study aimed to examine effective stress reduction interventions for nurses and to identify key elements of these successful interventions. Studies included in this review were conducted in acute psychiatric settings. A comprehensive search of electronic databases was conducted for support intervention studies between 2010 and 2021. The search yielded 315 studies that were reduced to seven studies after being reviewed by two independent reviewers. The studies were coded, and key elements were identified. Seven studies that were included consisted of a randomised controlled trial, quasi-experimental design and single-group design. Interventions included mindfulness-based stress reduction, burnout prevention programmes, communication skills, educational programme, group intervention, resilience training programme and stress management. Four key elements emerged from these interventions, namely, educational support, interpersonal skills, psychological support and adaptive coping. The findings highlighted the diverse interventions in supporting psychiatric nurses to cope with stress. However, there is a dearth of studies in acute psychiatric settings that were mostly done in emergency settings. Knowledge gained from this review may assist with practice improvement as managers can implement the identified interventions.

**Contribution:**

This is the first systematic review focusing on supportive interventions for nurses in acute psychiatric settings. The knowledge gained from this review will add to the existing research knowledge base in the field.

## Introduction

The psychiatric setting can be a stressful working environment for nurses caring for people with mental illness (Foster, Wood & Clowes 2020:2). Within this environment, patients admitted to acute psychiatric units compound the stress experienced by nurses as they are often admitted with unpredictable violent behaviour (Lozzino et al. 2015:15). Researchers have alluded to mental health consequences for nurses caring for patients presenting with violence that include burnout (Morse et al. 2012:344), poor mental and physical health (Kelly et al. 2016:711) and compromised well-being (Edward, Hercelinsyj & Giandinoto 2017:216).

Diverse psychosocial interventions are implemented to provide support for nurses working in psychiatric settings (Foster et al. 2018; Guay, Goncalves & Boyer 2016; Inoue, Kaneko & Okamura 2011). These interventions are aimed at modifying psychological and social factors (Ruddy & House 2005:3) as they either prevent or reduce the effects of stress to improve the psychological well-being of staff. The interventions include but not limited to mindfulness-based stress reduction (MBSR) programme (Kabat-Zinn 2003), mindfulness-based cognitive therapy (Segal, Williams & Teasdale 2013), resilience training programmes (Foster et al. 2018), educational programmes (Guay et al. 2016) and yoga programmes (Mandal et al. 2021).

Several studies (Brady et al. 2012; Guay et al. 2016; Sailaxmi & Lalitha 2015) explored the impact of psychosocial interventions on the mental health well-being of nurses working in psychiatric settings and have showed a positive impact. Mindfulness-based stress reduction interventions have showed positive results with various programmes offering brief time-limited interventions aimed at improving psychological well-being. A structured yoga programme that composed of 12 weeks of 20 sessions showed positive effects in reducing stress and improving quality of life among nursing staff (Mandal et al. 2021:8). Mindfulness-based stress reduction programmes have showed impact in reducing the levels of anxiety and depression (Yang, Tang & Zhou 2018:192) and perceived stress and burnout among psychiatric nurses (Edwards 2015:62). Studies on the effectiveness of stress management training programmes that are aimed at improving the psychological well-being of nurses showed positive effects of the interventions by improving coping strategies among nurses (Alkhawaldeh et al. 2019:130; Pahlevani et al. 2015:316).

A review by Foster et al. (2019:80) confirmed that resilience training programmes were beneficial for nurses working in mental health settings. While these interventions share common aims, which are, to reduce perceived stress, improve coping skills, as well as improve psychological well-being of nurses, they tend to differ, in terms of delivery and the duration of the programme. However, literature showed that there is a paucity of studies on supportive interventions conducted in acute psychiatric settings; studies were mostly conducted in emergency settings that motivated the researcher to conduct this systematic review to examine effective interventions in supporting nurses working in acute psychiatry and to identify aspects (key elements) within the interventions that were deemed effective. In this review, the researcher intends to compare and summarise findings from studies that address the range of supportive interventions for nurses working in acute psychiatric settings.

### Review objectives

The main objective of this review was to examine effective stress reduction interventions for supporting nurses exposed to patient violence in acute psychiatric units. The second objective was to identify the key elements of such interventions that support nurses to cope and improve their well-being.

### Review questions

- What are the stress reduction intervention programmes for nurses exposed to patient violence in acute psychiatric units?
- What are the key elements of these effective interventions?

## Method

This review was conducted using standard systematic review methodology following the Preferred Reporting Items for Systematic Reviews and Meta-Analysis Protocols (PRISMA-P) 2015 statement (Moher et al. 2015).

### Eligibility criteria

#### Inclusion and exclusion criteria

Articles were only considered for inclusion if they included nurses (professional and non-professional) working in acute psychiatric units, as well as general hospitals. Studies were also eligible if the population involved other healthcare workers, which included nurses as part of the sample. Studies had to be published between 2010 and 2021; the literature showed that most studies that focussed on psychological interventions were conducted during this time. Studies had to be written in English. Studies that aimed at providing formal and informal, individual or group support to nurses working in acute care were also included. Only studies that were conducted in acute psychiatric units in both general and psychiatric hospitals were included.

All study designs were considered published in peer-reviewed journals, randomised controlled trials, quasi-experimental studies, systematic reviews or evaluation studies aimed at providing support for nurses. However, studies with the following criteria were excluded: non-English, if nurses were not included in the population, setting was not an acute psychiatric care unit, scoping reviews, narrative reviews, literature reviews and protocols.

### Search strategy

The search strategy was aimed at locating published, as well as unpublished studies. The researcher conducted a search on Medical Literature Analysis and Retrieval System Online (MEDLINE) and Cumulative Index for Nursing and Allied Health Literature (CINAHL) to identify articles related to the topic. The following multiple search terms, and any other synonyms, in combination with Boolean operators, were used in the search of all databases, including (intervention OR ‘intervention programme’ OR ‘intervention strategy’ OR ‘supportive intervention’ OR ‘staff support programme’ OR ‘programme’) AND (emotional support). AND (‘acute mental health’ OR ‘psychiatric ward’ OR ‘psychiatric hospital’ OR ‘mental health unit’) AND (nurs* OR ‘nurses’ OR ‘nursing’). A comprehensive search was done from the following databases: Academic Search Complete, CINAHL , Google Scholar, Health Source: Nursing/Academic Edition (EbscoHost) MEDLINE, PubMed and Wiley Online Library. The researcher screened the reference list of all identified articles for possible additional studies that could have been missed during the search. The search generated 315 articles.

### Critical appraisal

The two independent reviewers (N.B. and P.M.) critically appraised the seven selected articles, for methodological quality, using the following standardised instruments: The Johanna Briggs Institute (JBI) critical appraisal checklist for Randomised Controlled Trials and JBI critical appraisal checklist for quasi-experimental studies. Quality assessment was performed by reviewers independently (N.B. and P.M.) (Table 1 and Table 2). The reviewers discussed any disagreements to reach consensus or third reviewer was involved.

**TABLE 1:** Quality score for quasi-experimental studies using JBI appraisal checklist for quasi-experimental studies.

Author and year	Q1	Q2	Q3	Q4	Q5	Q6	Q7	Q8	Q9	Score	Quality
1. Chen et al. (2010)	Y	N	N	Y	Y	Y	Y	Y	Y	7	Moderate
2. Sailaxmi and Lalitha (2015)	Y	Y	N	N	Y	Y	NA	Y	Y	6	Moderate
3. Alenezi, McAndrew and Fallon (2019)	Y	Y	N	Y	Y	U	Y	Y	Y	7	Moderate
4. Ghazavi, Lohrasbi and Mehrabi (2010)	Y	Y	N	Y	Y	Y	Y	Y	Y	8	High
5. Foster et al. (2018)	Y	Y	N	N	Y	NA	NA	Y	Y	4	Low

*Source*: Adapted from Mansoor, K. & Khuwaja, H.M.A., 2020, ‘The effectiveness of a chronic disease self-management program for elderly people: a systematic review’, *Elderly Health Journal* 6(1), 51–63

Y, Yes; N, No; U, Unclear; NA, Not Applicable; Q, Question.

Score grading: (1–4 low); 5–7 (moderate); 8–9 (high).

**TABLE 2:** Quality score of randomised controlled trials using JBI appraisal checklist for randomised controlled trial.

Author and year	Q1	Q2	Q3	Q4	Q5	Q6	Q7	Q8	Q9	Q10	Q11	Q12	Score	Quality
Yang, Tang and Zhou (2019)	Y	Y	Y	Y	Y	U	Y	Y	Y	Y	Y	Y	11	High
Inoue, Kaneko and Okamura (2011)	Y	Y	Y	Y	Y	U	Y	Y	Y	Y	Y	Y	11	High

*Source*: Adapted from Mansoor, K. & Khuwaja, H.M.A., 2020, ‘The effectiveness of a chronic disease self-management program for elderly people: a systematic review’, *Elderly Health Journal* 6(1), 51–63

Y, Yes, N, No, U, Unclear, Q, Question.

Score grading: 1–5 low; 6–8 moderate; 9–12 high.

### Data extraction

Two independent reviewers (N.B. and P.M.) extracted data for the review. The following information was extracted: authors, year, country, design, intervention, setting, sample, outcome scales, and findings. The reviewers resolved disagreements through discussion or with the involvement of a third reviewer.

### Data synthesis and reporting

A textual narrative qualitative data synthesis was undertaken for the extracted data because of the heterogeneity of the interventions and outcomes measured. This type of analysis is suitable for synthesizing evidence of different studies (Barnett-Page & Thomas 2009). The PRISMA statement was used as a guideline to report the systematic reviews of these interventions.

### Ethical considerations

Ethical clearance was obtained from Biomedical Science Research Ethics Committee (reference number: BM18/6/8) of the University of the Western Cape, this systematic review forms part of the process towards completion of the PhD.

## Results

### Study selection

**FIGURE 1:**
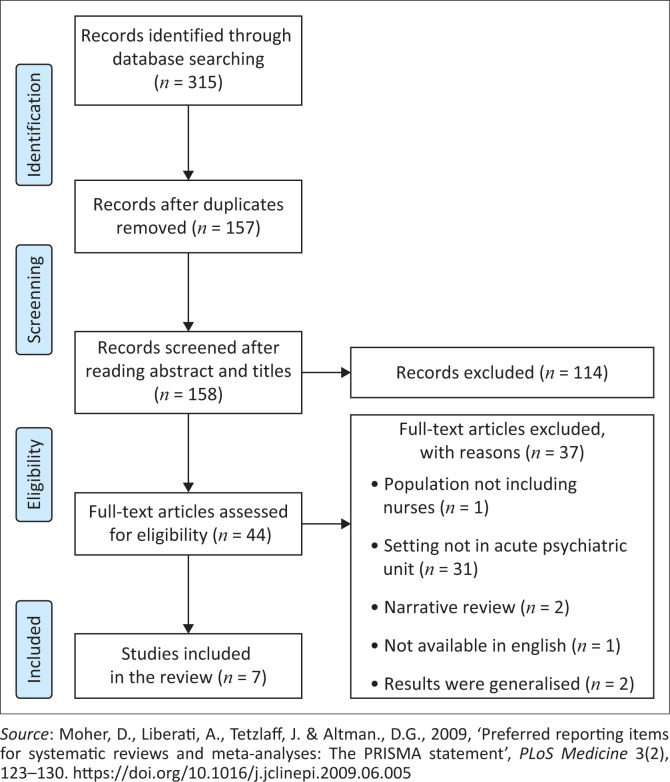
Preferred reporting items for systematic reviews, and meta-analysis 2009 flow diagram detailing the search and selection process of studies included in the review.

A total of 315 articles were generated from all databases searched. All identified articles were collated and imported to reference manager software Endnote X7.8, which automatically identified duplicates. Following the removal of 157 duplicates, 158 articles remained for the screening process of the titles and abstracts, against the inclusion criteria, which was undertaken by two independent reviewers (N.B. and P.M.). A total of 114 articles were found not relevant for the review based on the title and abstract screening and were excluded. The remaining 44 articles were retrieved and reviewed by two independent reviewers (N.B. and P.M.), against the inclusion criteria to make the final decision whether the article will be included in the review, and a further 37 articles were excluded. Reasons for the exclusion of articles were recorded and are displayed in Figure 1. There were seven articles that were deemed suitable for inclusion in the review. Disagreements between the reviewers were resolved through discussion. Figure 1 outlines the search process, results and reasons for the exclusion of articles.

### Characteristics of the included studies

The seven included studies were conducted between 2010 and 2019 in various countries (Table 3). All studies were conducted in acute psychiatric care settings. The number of recruited participants in the studies ranged from 16 to 296. The included studies consisted of two randomised controlled trials, four quasi-experimental studies and one single group pre-test–post-test design. The identified interventions included MBSR, a burnout prevention programme, a communication skills programme, an education and training programme, a group intervention programme, a resilience training programme and a stress management programme that were reported as being effective in improving psychological well-being.

**TABLE 3:** Summary of selected studies for review.

Authors/year/country	Design	Intervention	Setting	Sample	Outcome scales	Findings
Chen et al. (2010), Taiwan	Quasi-experimental research design Quality: High	Education programme: 43.5-h-long lessons (8-week). (1) Potentiality & intention and cultivation of creativity; (2) intention and cultivation of absorption and organisation ability; (3) intention and cultivation of stability and coping ability; and (4) intention and cultivation of control ability and potentiality.	Three psychiatric hospitals in Taipei, Taoyuan and Hualien	Fifty-nine psychiatric nurses from three psychiatric hospitals in Taipei IG = 26 CG = 33	Lee’s Index of Work Satisfaction (IWS) Williams’ (1980) Creativity Assessment Packet and clinical expertise.	Job satisfaction: IG 3.33↑ versus CG3.16 Potentiality: IG3.22↔CG3.15
Inoue et al. (2011), Japan	Randomised controlled trial Quality: High	Group intervention approach programme: Program composed-psychotherapy based discussion, including topics on coping with violent speech, violence or psychological impacts and stress management, behavioural therapy. Duration: four sessions weekly for 4 weeks, 90 min	Five psychiatric hospitals in the Chugoku and Kyushu district – acute psychiatric care and chronic psychiatric care	Sixty-two psychiatric nurses IG = 30 CG = 32	Impact of Event Scale Revised (IES-R) Profile Mood State (POMS)	At baseline: IG ↔CG Immediately after intervention: IG 4.91 ↑CG 3.65 1 month after intervention: IG 5.21 ↑CG 5.00 Immediately after intervention: IG 4.95 ↑CG 5.23 1 month after intervention: IG 4.16 ↑CG 6.10
Foster et al. (2018), Australia	Single group pre-test-post-test design Quality: Low	Promoting adult resilience programme: Seven modules delivered face to face, weekly. Components include identifying strengths and understanding resilience, understanding and managing stress, challenging and changing negative self-talk, drawing strength from adversity, promoting positive relationships, managing conflict, creating solutions for well-being.	Two acute adult inpatient units	Twenty-four registered nurses	DASS 21 Scale Satisfaction with Life Scale and Ryff’s Scale of Psychological Well-being	Low levels of stress were observed 3 months after the programme
Sailaxmi and Lalitha (2015), India	Quasi-experimental one group pretest-posttest design Quality: Moderate	Stress management programme Ten consecutive, 1 h sessions Five sessions in a week for 2 weeks Sessions focused on stress education, problem solving, time management, taking time off, communication skills, assertiveness training, responding to criticism, negotiation skills and humour.	Psychiatric hospital at Bangalore Psychiatry special wards, emergency unit, closed psychiatry wards and open psychiatry wards.	Fifty-three nurses	The DCL Stress scale (The De Villiers, Carson & Leary Stress Scale; Carson et al. 1995)	Pre-intervention 57.45 Immediately following intervention ↓41.06 Four weeks after the intervention. ↓26.43
Yang et al. (2018), China	Randomised controlled trial Quality: High	Mindfulness-based stress reduction therapy (MBSR) Once a week for 8 weeks. Practised at home or during the session. First stage, relaxation preparation – rest posture using relaxing Chinese music; Second stage, mindfulness breathing Third stage-mind-fulness meditation,	Three general hospitals in Hunan province of China	Hundred psychiatric nurses IG = 50 CG = 50	Symptom Checklist-90 (SCL-90) scale, Self-Rating Depression Scale (SDS), Self-Rating Anxiety Scale (SAS), Nursing Stress Scale.	SCL-90 Before IG 136.7↔ CG 134.5 After IG 119.6↓ CG 132.6 Self-Rating Depression Scale (SDS), Before IG 45.8↔ CG 43.3 After IG 35.4 ↓ CG 41.2 Self-Rating Anxiety Scale (SAS), Before IG 44.8 ↔CG 46.2 After IG 36.4↓ CG 45.1 Nursing Stress Scale. Before IG 83.9 ↔ CG 84.8 After IG 68.2↓ CG 83.1
Alenezi et al. (2019), Saudi Arabia	Quasi-experimental study utilising non-equivalent pretest-posttest design Quality: Moderate	Burnout prevention workshop Education on burnout: Tips for creating space for relaxation; Self-care activities Stress reduction management; Progressive muscle relaxation; Social skill training; Communication skills training. Intervention delivered over 2 days’ (6 h per day) three group running simultaneously	Al Amal Complex for Mental Health in Riyadh.	Two hundred and ninety six nurses IG = 154 CG = 142	Maslach Burnout Inventory (MBI) measured the effects of the workshop at 1-, 3- and 6-month intervals after completion of the programme.	Prior intervention- total burnout score IG 71.13↑ CG 66.28 1-month post intervention IG 63.15 CG 67.93↑ 3-month post intervention IG 64.88 CG 68.74↑ 6-month post intervention IG 66.15 CG 69.99↑
Ghazavi et al. (2010), Iran	Quasi-experimental study design Quality: High	Communication skills training Group psychoeducation Six sessions in 3 weeks – delivering lecture, problem solving, brain storming, sharing the experiences of the members and discussion and using personal computers and whiteboard 1.5 h per session	Active psychiatry wards. emergency or acute, chronic, or specialised men and women ward of psychiatry in two psychiatric hospitals	Forty-five psychiatric nurses IG 23 CG 22	Researcher designed questionnaire based on Tuft-Anderson’s questionnaire, psychiatric nurses occupational stress scale (PNOSS) before, after and 1 month after the intervention.	Before intervention IG 63.3↔ CG 63.2 Immediately after intervention IG 54.9 ↓ versus CG 63.9 One month after intervention IG 54.8↓ versus CG 64.3

*Source*: Adapted from Gilbertson-White, S., Saeidzadeh, S., Yeung, C.W., Tykol, H. & Vikas, P., 2017, ‘Palliative and supportive interventions to improve patient-reported outcomes in rural residents with cancer,’ *The Journal of Community and Supportive Oncology*. https://doi.org/10.12788/jcso.0348

IG, Intervention Group; CG, Control Group; ↔, no significant difference; ↑, significant increase; ↓, significant decrease.

The second objective of this review was to identify key elements of the effective interventions. There were four key elements that emerged from the analysis of the interventions of the included studies: educational support, interpersonal skills, psychological support and adaptive coping.

#### Educational support

Most studies used educational sessions as a mode of delivery of the intervention; these sessions were facilitated by trained professionals. Providing educational sessions in group settings were effective elements of interventions to impart knowledge among participants about healthy ways of dealing with stress related to the exposure to violent behaviour displayed by patients in the workplace. The education programme aimed to ‘assist psychiatric nurses in determining their abilities and be able to identify what hinders them from developing and improving their job satisfaction’ (Chen et al. 2010:88). Education on potentiality has demonstrated to have increased chances of an individual to achieve their potentiality. In Sailaxmi and Lalitha (2015:4) and Yang et al. (2018:191), participants from their respective studies were encouraged to share their experiences among each other in the group in a form of case scenarios and have robust discussions in which they each learnt from each other’s experiences. These discussions included stress management techniques that were being used by individuals to cope with stress. The findings showed a significant reduction in nurses’ perceived stress levels following the intervention, which indicate that these stress management techniques were feasible. A study by Inoue et al. (2011:6) aimed at providing support for nurses exposed to violence to improve their mental health. The results showed that by sharing knowledge in group discussions, nurses gained more confidence on how to handle future violent incidents, and intervention had a positive effect in alleviating stress.

#### Interpersonal skills

Ghazavi, Lohrasbi and Mehrabi (2010:399) identified communication skills as necessary to assist nurses to become more aware of their communication errors when interacting with patients. They also suggested that there should be regular educational training on communication. The results showed that teaching communication skills using the psychoeducation method decreases perceived stress for nurses working in psychiatric wards (Ghazavi et al. 2010:399). Communication breakdown between nurses and patients can result in exposure to violent incidents.

#### Psychological support

Inoue et al. (2011:6) discuss various measures of dealing with violence in a psychological group setting. This type of group allowed participants an opportunity to handle the after-effects of being exposed to violent incidents, their emotions and to manage stress. It created a conducive environment where nurses could speak freely and share the negative or positive experience. The results showed that participants had gained confidence in dealing with their feelings following a violent incident. Following the implementation of the intervention, the psychotherapy group had an effect on participants’ anxiety about possible violent incidents and the depression that results from such anxiety seemed to have been alleviated. A study conducted by Yang et al. (2018:190) implemented psychological intervention to provide support to psychiatric nurses. The service included MBSR, which was divided into mindfulness meditation, relaxation and mindfulness breathing exercise. This has been shown to improve mental health and well-being of psychiatric nurses (Yang et al. 2018:193).

#### Adaptive coping

Studies by Chen et al. (2010) and Inoue et al. (2011) alluded to the content of educational sessions held that included discussions on coping strategies in dealing with violence from psychiatric patients and the impact on nurses’ psychological well-being. Various strategies were discussed in groups and yielded positive results in terms of alleviating any symptoms that participants were suffering from prior to the implementation of the intervention (Inoue et al. 2011). Coping ability is seen as an important aspect of potentiality education (Chen et al. 2010). Problem-solving skills are regarded as an important element for nurses to be able to cope with stressful situations in their working environment (Ghazavi et al. 2010; Sailaxmi & Lalitha 2015). Results showed a significant impact on stress reduction and how nurses cope with challenges using positive coping mechanisms (Sailaxmi & Lalitha 2015:3).

## Discussion

The aim of this systematic review was to examine effective stress reduction interventions to support nurses exposed to violent incidents and to identify key elements of these interventions. The focus of the interventions included in this review was not on violence exhibited by the patient but rather on support provided for nurses working in acute psychiatric settings. Some of the studies examined the effect of MBSR programmes and group psychotherapy in reducing perceived stress of nurses who experienced violence in the workplace. The current review found that mindfulness meditation, relaxation techniques and mindfulness exercises significantly reduce perceived stress and burnout among psychiatric nurses (Inoue et al. 2011:6; Yang et al. 2018). This is consistent with a previous study that reported a decrease in stress levels, reduction in burnout symptoms and depressed mood (Craigie et al. 2016:770). Guillaumie, Boiral and Champagne (2017:1028) reported that support intervention improved psychological well-being and performance of nurses at work. Cohen-Katz et al. (2005a, 2005b), Raingrunber & Robinson (2007), Richards et al. (2006) in Guillaumie et al. (2017:1023) state that mindfulness facilitated a state of calmness and communication with patients, mainly because it helps participants maintain emotional balance and experience less frustration and anger at work. On the other hand, a study by Watanabe et al. (2019:190) showed no significant effects of the MBSR, stating possible reasons as may be the duration and timing of a programme, training being administered by untrained therapists and participants possibly having been resilient.

Four key elements were identified in the analysis of the seven selected studies. Educational support was widely used in most studies as a mode of sharing knowledge and experience with participants, facilitated by trained professionals who have knowledge of the phenomena of interest. McDonald et al. (2012:382) state that trained facilitators played a huge role during group sessions as they become teacher, challenger, encourager, nurturer and motivator. Providing educational sessions in group settings was an effective element of interventions to facilitate sharing of information on ways of coping with stressful situations in the workplace. It empowers participants with knowledge that helped them control and overcome any negative thoughts (Foster et al. 2018:1477). The findings of this review are consistent with other studies that employed educational sessions in the form of a workshop as a means of disseminating information among groups and have shown an effect on staff confidence in coping with stressful situations in the workplace (Lamont & Brunero 2018; McDonald et al. 2012:382).

Sharing of experiences during group sessions allowed nurses an opportunity to learn from one another and is viewed as an advantage. Group discussion is a method whereby participants have a platform to express, present and argue their knowledge, experiences, opinions and feelings (Rahman et al. 2011). It gives them an opportunity to respond to others’ ideas while reflecting on their own in an effort to build their knowledge and understanding of the matter at hand. Findings from a pilot study on the effectiveness of psychotherapeutic groups where participants shared experiences showed a reduced risk of burnout and became more aware of their emotions (Floriana, Luca & Simona 2016:61).

Effective communication skills were deemed the most important aspects of the interventions in empowering psychiatric nurses, as they spent most of their time interacting with patients. It plays a role in building nurse–patient relationship and creating an understanding between a nurse and a patient (Alshammari, Duff & Guilhermino 2019:2; Yao et al. 2021:178). A study by Ghazavi et al. (2010:399) demonstrated that improved communication skills of nurses successfully reduced stress levels and that was sustained for a month after the training. In addition, participants in their study were doing better in communication following the implementation of a communication-based group intervention (Baby, Gale & Swain 2019:177). Furthermore, a review by Tolli et al. (2017:2821) showed that training interventions were more likely to increase the confidence of the staff in managing violent incidents and enhance their communication skills.

Current findings show that discussions about adaptive coping strategies among nurses yielded positive results in empowering nurses on ways of coping with stressful situations in their workplace. The results showed a significant decrease in perceived stress and improved coping skills (Ghazavi et al. 2010; Sailaxmi & Lalitha 2015:3). Findings from a study by Foster et al. (2018:1477) indicated improved coping self-efficiency of nurses, decreased mental distress and improved cognitive and behavioural resilience strategies. Similarly, Mandal et al. (2021) evaluated the effect of a yoga programme in reducing perceived stress and improving coping skills and revealed that yoga sessions led to increased coping ability and also had a positive effect on stress reduction.

### Limitation of the study

The limitation of this review includes its inclusion criteria that only focuses on published studies on psychosocial interventions for nurses in acute psychiatric settings. It is possible that more literature exists on interventions meant to provide support for nurses in psychiatry. Studies limited to the English language only could limit access to more information that is published in other languages. Lastly, the lack of control group in some of the studies to compare results should be viewed with caution.

## Conclusion

This systematic review highlighted diverse psychosocial interventions with different practices in providing support to psychiatric nurses working in stressful environments that have the potential of leading to adverse psychological consequences for nurses. However, there were fewer studies that met the inclusion criteria of the review, which were done in acute psychiatric care; most studies that were excluded were done in emergency settings. Four key elements of successful interventions to support nurses exposed to violent incidents were identified. Diverse psychological interventions utilised educational group sessions which increased the understanding of participants about challenges in their work environment and how to cope in a positive way with these challenges that impacted positively on their mental health, well-being and job satisfaction. The knowledge gained from this review may assist with practice improvement as managers can implement some of the interventions identified to support nurses.

### Implications of the study

Nurses working in acute psychiatric settings face many challenges when providing care for patients. The findings of this review highlighted the positive impact of these psychosocial interventions by providing support for the nurses working in acute psychiatric settings. The knowledge gained from this review will add to the existing research knowledge base in the field. The interventions identified for this review were only focused on support and not on nurses’ exposure to violence in acute psychiatric settings. Future research should be conducted, which will focus on nurses’ exposure to violent incidents while caring for patients and its impact on their well-being.

## References

[CIT0001] Alenezi, A., McAndrew, S. & Fallon, P., 2019, ‘Burning out physical and emotional fatigue: Evaluating the effects of a programme aimed at reducing burnout among mental health nurses’, *International Journal of Mental Health Nursing* 28(5), 1045–1055. 10.1111/inm.12608 31231965

[CIT0002] Alkhawaldeh, J.M., Soh, K.L., Mukhtar, F., Peng, O.C, Alkhawaldeh, H.M., Al-Amer, R. et al., 2019, ‘Stress management training for stress reduction and coping improvement in public health nurse: A randomized controlled trial’, *Journal of Advanced Nursing* 76(11), 3123–3135. 10.1111/jan.14506 32856353

[CIT0003] Alshammari, M., Duff, J. & Guilhermino, M., 2019, ‘Barriers to nurse-patient communication in Saudi Arabia: An integrative review’, *BMC Nursing* 18, 61. 10.1186/s12912-019-0385-4 31827387 PMC6892156

[CIT0004] Baby, M., Gale, C. & Swain, N., 2019, ‘A communication skills intervention to minimise patient perpetrated aggression for healthcare support workers in New Zealand: A cluster randomised controlled trial’, *Health and Social Care in the Community* 27(1), 170–181. 10.1111/hsc.12636 30175538

[CIT0005] Barnett-Page, E. & Thomas, J., 2009, ‘Methods for the synthesis of qualitative research: A critical review’, *BMC Medical Research Methodology* 9, 59. 10.1186/1471-2288-9-59 19671152 PMC3224695

[CIT0006] Brady, S., O’Connor, N., Burgermeister, D. & Hanson, P., 2012, ‘The impact of mindfulness meditation in promoting a culture of safety on an acute psychiatric unit’, *Perspective in Psychiatric Care* 48(3), 129–137. 10.1111/j.1744-6163.2011.00315.x 22724398

[CIT0007] Carson, J., Leary, J., De Villiers, N., Fagin, L. & Radmall, J., 1995, ‘Stress in mental health nurses: comparison of wards and community staff’, *British Journal of Nursing* 7(4), 579–582. 10.12968/bjon.1995.4.10.579 7599484

[CIT0008] Chen, K.H., Lee, S., Weng, L.C. & Chen, Y.J., 2010, ‘The effects of potentiality education on potentiality and job satisfaction among psychiatric nurses in Taiwan’, *Perspectives in Psychiatric Care* 46(2), 85–97. 10.1111/j.1744-6163.2010.00244.x 20377796

[CIT0009] Craigie, M., Slayter, S., Hegney, D., Osseiran-Moisson, R., Gentry, E., Davis, S. et al., 2016, ‘A pilot evaluation of a mindful self-care and resiliency (MSCR) intervention for nurses’, *Mindfulness* 7, 764–774. 10.1007/s12671-016-0516-x

[CIT0010] Cohen-Katz, J., Wiley, S. D., Capuano, T., Baker, D.M., Deitrick, L. & Shapiro, S., 2005a, ‘The effects mindfulness-based stress reduction on nurses stress and burnout: A qualitative and quantitative study, part III’, *Holistic Nursing Practice* 19(2), 78–86. 15871591 10.1097/00004650-200503000-00009

[CIT0011] Cohen-Katz, J., Wiley, S.D., Capuano, T., Baker, D.M., Kimmel, S. & Shapiro, S., 2005b, ‘The effects of mindfulness-based stress reduction on nurse stress and burnout, part II: A quantitative and qualitative study’, *Holistic Nursing Practice* 19, 26–35. 15736727 10.1097/00004650-200501000-00008

[CIT0012] Edward, K., Hercelinsyj, G. & Giandinoto, J., 2017, ‘Emotional labour in mental health nursing: An integrative systematic review’, *International Journal of Mental Health Nursing* 26(3), 215–225. 10.1111/inm.12330 28374961

[CIT0013] Edwards, L.W.B., 2015, ‘A mindfulness and health promotion program to decrease the perception of stress and burnout in psychiatric mental health nurses who provide direct patient care to individuals in mental health units with a diagnosis of Alzheimer’s type dementia’, PhD thesis, University of Southern Mississippi.

[CIT0014] Floriana, C., Luca, C. & Simona, G., 2019, ‘Effectiveness of a short-term psychotherapeutic group for doctors and nurses in a hospice in Southern Europe’, *Progress in Palliative Care* 27(2), 58–63. 10.1080/09699260.2019.1612136

[CIT0015] Foster, K., Roche, M., Delgado, C., Cuzzillo, C., Giandinoto, J.A. & Furness, T., 2019, ‘Resilience, and mental health nursing: An integrative review of international literature’, *International Journal of Mental Health Nursing* 28(1), 71–85. 10.1111/inm.12548 30294937

[CIT0016] Foster, K., Sochet, I., Wurfl, A., Maybery, D., Shakespeare-Finch, J. & Furness, T., 2018, ‘On PAR: A feasibility study of the promoting adult resilience programme with mental health nurses’, *International Journal of Mental Health Nursing* 27(5), 1470–1480. 10.1111/inm.12447 29488298

[CIT0017] Foster, A., Wood, E. & Clowes, M., 2020, ‘Identifying the evidence base of interventions supporting mental health nurses to cope with stressful working environment: A scoping review’, *Journal of Nursing Management* 29(6), 1639–1652. 10.1111/jonm.13312 33742495

[CIT0018] Ghazavi, Z., Lohrasbi, F. & Mehrabi, T, 2010, ‘Effect of communication skills training using psychoeducation method on the stress levels of psychiatry ward nurses’, *Iranian Journal of Nursing and Midwifery Research* 15(Suppl 1), 395–400. 22069416 PMC3208939

[CIT0019] Gilbertson-White, S., Saeidzadeh, S., Yeung, C.W., Tykol, H. & Vikas, P., 2017, ‘Palliative and supportive interventions to improve patient-reported outcomes in rural residents with cancer’, *The Journal of Community and Supportive Oncology*. 10.12788/jcso.0348

[CIT0020] Guay, S., Goncalves, J. & Boyer, R., 2016, ‘Evaluation of an education and training program to prevent and manage patients’ violence in a mental health setting: A pretest-posttest intervention study’, *Healthcare* 4(3), 49. 10.3390/healthcare4030049 PMC5041050 27490582

[CIT0021] Guillaumie, L., Boiral, O. & Champagne, J., 2017, ‘A mixed methods systematic review of the effects of mindfulness on nurses’, *Journal of Advanced Nursing* 73(5), 1017–1034. 10.1111/jan.13176 27706912

[CIT0022] Inoue, M., Kaneko, F. & Okamura, H., 2011, ‘Evaluation of a group intervention approach for nurses exposed to violent speech or violence caused by patients: A randomised controlled trial’, *International Scholarly Research Network* 2011, 325614. 10.5402/2011/325614 PMC3169854 21994892

[CIT0023] Kabat-Zinn, J., 2003, ‘Mindfulness-based interventions in context: Past, present and future’, *Clinical Psychology: Science and Practice* 10(2), 144–156. 10.1093/clipsy.bpg016

[CIT0024] Kelly, E.L., Fernwick, K., Brekke, J.S. & Novaco, R.W., 2016, ‘Well-being and safety among inpatient psychiatric staff: The impact of conflict, assault, and stress reactivity’, *Administration and Policy in Mental Health* 43(5), 703–716. 10.1007/s10488-015-0683-4 26377816 PMC4794422

[CIT0025] Lamont, S. & Brunero, S., 2018, ‘The effect of workplace violence training program for generalist nurses in the acute hospital setting: A quasi-experimental study’, *Nurse Education Today*, 68, 45–52. 10.1016/j.nedt.2018.05.008 29885569

[CIT0026] Lozzino, L., Ferrari, C., Large, M., Nidssen, O. & De Girolamo, G., 2015, ‘Prevalence, and risk factors of violence by psychiatric acute inpatients: Systematic review and meta-analysis’, *PLoS One* 10(6), 1–18. 10.1371/journal.pone.0128536 PMC4464653 26061796

[CIT0027] Mandal, S., Misra, P., Sharma, G., Sagar, R., Kant, S., Dwivedi, S.N. et al., 2021, ‘Effect of structured yoga program on stress and professional quality of life among nursing staff in a tertiary care hospital of Dehli: A small -scale phase -II trial’, *Journal of Evidence-based Integrative Medicine* 26, 1–10. 10.1177/2515690X21991998 PMC7882766 33567888

[CIT0028] Mansoor, K. & Khuwaja, H.M.A., 2020, ‘The effectiveness of a chronic disease self-management program for elderly people: a systematic review’, *Elderly Health Journal*, 6(1), 51–63.

[CIT0029] McDonald, G., Jackson, D., Wilkes, L. & Vickers, M.H., 2012, ‘A work-based educational intervention to support the development of personal resilience in nurses and midwives’, *Nurse Education Today* 32(4), 378–384. 10.1016/j.nedt.2011.04.012 21724307

[CIT0030] Morse, G., Salyers, M., Rollins, A., Monroe-DeVita, M. & Pfahler, C., 2012, ‘Burnout in mental health services: A review of the problem and its remediation’, *Administration and Policy in Mental Health* 39(5), 341–352. 10.100/s10488-011-0352-1 21533847 PMC3156844

[CIT0031] Moher, D., Liberati, A., Tetzlaff, J. & Altman, D.G., 2009, ‘Preferred reporting items for systematic reviews and meta-analyses: The PRISMA statement’, *PLoS Medicine* 3(2), 123–130. 10.1016/j.jclinepi.2009.06.005 PMC3090117 21603045

[CIT0032] Moher, D., Shamseer, L., Clarke, M., Ghersi, D., Liberati, A., Petticrew, M. et al., 2015, ‘Preferred reporting items for systematic review and meta-analysis protocols (PRISMA-P) 2015 statement’, *Systematic Review* 4, 1. 10.1186/2046-4053-4-1 PMC4320440 25554246

[CIT0033] Pahlevani, M., Ebrahim, M., Radmehr, S., Amini, F., Bahraminasab, M. & Yazdani, M., 2015, ‘Effectiveness of stress management training on the psychological well-being of the nurses’, *Journal of Medicine and Life* 8(4), 313–318. 28316750 PMC5319283

[CIT0034] Rahman, F., Khalil, J.K., Jumani, N.B., Ajmal, M., Malik, S. & Sharif, M, 2011, ‘Impact of discussion method on student performance’, *International Journal of Business and Social Science* 2(7).

[CIT0035] Rangruber, B. & Robinson, C., 2007, ‘The effectiveness of Tai Chi, Yoga, Meditation and Reiki healing sessions in promoting health and enhancing problem solving abilities of registered nurses’, *Issues in Mental Health Nursing* 28(10), 1141–1155. 17957554 10.1080/01612840701581255

[CIT0036] Richards, J.A., Oman, D., Hedberg, J., Thoresen, C.E. & Bowden, J, 2006, ‘A qualitative examination of a spirituality-based intervention and self-management in the workplace’, *Nursing Science Quarterly* 19(3), 231–239. 16757790 10.1177/0894318406289490

[CIT0037] Ruddy, R. & House, A., 2005, ‘Psychosocial interventions for conversion disorder’, *CochraneDatabase Systematic Review* (4), 1–27. 10.1002/14651858.CD005331.pub2 16235402

[CIT0038] Sailaxmi, G. & Lalitha, K., 2015, ‘Impact of a stress management program on stress perception of nurses working with psychiatric patients’, *Asian Journal of Psychiatry* 14, 42–45. 10.1016/j.ajp.2015.01.002 25703040

[CIT0039] Segal, Z.V., Williams, J.M.G. & Teasdale, J.D., 2002, *Mindfulness-based cognitive therapy for depression: A new approach to preventing relapse*, Guilford Press, New York, NY.

[CIT0040] Tolli, S., Partanen, P., Kantio, R. & Haggma-Latila, A., 2017, ‘A quantitative systematic review of the effects of training interventions on enhancing the competence of nursing staff in managing challenging patient behaviour’, *Journal of Advanced Nursing* 73(12), 2817–2831. 10.1111/jan.13351 28556934

[CIT0041] Watanabe, N., Horikoshi, M., Shinmei, I., Oe, Y., Narisawa, T., Kumachi, M. et al., 2019, ‘Brief mindfulness-based stress management program for a better mental state in working population- Happy nurse project: A randomised controlled trial’, *Journal of Affective Disorder* 251, 186–196. 10.1016/j.jad.2019.03.067 30927579

[CIT0042] Williams, F.E., 1980, *Creativity assessment packet (CAP): Manual*, D.O.K, Buffalo, New York.

[CIT0043] Yang, J., Tang, S. & Zhou, W., 2018, ‘Effect of mindfulness-based stress reduction therapy on work stress and mental health of psychiatric nurses’, *Psychiatria Danubina* 30(2), 186–196. https://doi.org/10.24869.psyd.2018.189 10.24869/psyd.2018.189 29930229

[CIT0044] Yao, X., Shao, J., Wang, L., Zhang, J., Zhang, C. & Lin, Y., 2021, ‘Does workplace violence, empathy, and communication influence occupational stress among mental health nurses?’, *International Journal of Mental Health Nursing* 30(1), 177–188. 10.1111/inm.12770 32808483

